# SYNCHRONIZE: Real-World Retrospective Safety Analysis of Patients Treated with OnabotulinumtoxinA for More than One Therapeutic Indication

**DOI:** 10.3390/toxins16100420

**Published:** 2024-09-29

**Authors:** Grace Forde, Benjamin M. Brucker, Kimberly Becker Ifantides, Atul T. Patel, Angeli Mayadev, Theodore Brown, Ziyad Ayyoub, Kenneth Martinez, Ritu Singh, Mariana Nelson, Simona Battucci, Irina Yushmanova, Ahunna Ukah, Christopher Rhyne

**Affiliations:** 1 NeuroPain Care Center, Lake Success, NY 11042, USA; 2 NYU Langone Health, New York, NY 10017, USA; 3 AbbVie, Irvine, CA 92612, USA; 4 Kansas City Bone & Joint Clinic, Overland Park, KS 66211, USA; 5 Swedish Neuroscience Institute, Seattle, WA 98122, USA; 6 EvergreenHealth Kirkland, Kirkland, WA 98034, USA; 7 Ranchos Los Amigos National Rehabilitation Center, Downey, CA 90242, USA; 8 Neurology & Pain Specialty Center, Aliso Viejo, CA 92656, USA; 9 AbbVie, 00144 Rome, Italy; 10 Diamond Headache Clinic, Chicago, IL 60642, USA

**Keywords:** chronic migraine, cervical dystonia, multiple therapeutic indications, onabotulinumtoxinA, overactive bladder, safety, spasticity, treatment-emergent adverse event

## Abstract

OnabotulinumtoxinA (onabotA) is approved in the US for 12 therapeutic indications. Real-world data on onabotA multi-indication use are limited, often leading to delayed or reduced treatment. This study provides real-world evidence on the safety of onabotA when treating multiple indications concomitantly. SYNCHRONIZE was a multicenter, retrospective, chart-review study evaluating onabotA’s safety for adults treated for ≥2 therapeutic indications within a 3-month period. The primary outcome was treatment-emergent adverse events (TEAEs) within 6 months post-treatment. A total of 279 patients were included. The most common concomitant indications treated were cervical dystonia and chronic migraine (43.4%). The average 3-month cumulative dose for multiple indications was 282.2 U. The treatment interval for multiple indications was ≤24 h for most patients (62.4%). Overall, 28.7% of patients reported ≥1 TEAE with no apparent trends in TEAEs and dose interval or cumulative dose. Reported TEAEs included UTI (5.7%), neck pain (5.0%), and headache (4.3%). No patient had a lack of effect according to clinical objective measurements. SYNCHRONIZE described the real-world safety of onabotA for patients treated concomitantly for ≥2 indications within a 3-month period. TEAEs were generally consistent with the known safety profiles of individual indications. No new safety signals were identified).

## 1. Introduction

OnabotulinumtoxinA (onabotA) is a purified botulinum toxin type A (BoNT/A) that is focally administered. It is a neuromodulator that cleaves synaptosomally associated protein (SNAP-25) at the terminal membrane of motor, autonomic, and sensory nerves, leading to reduced activity of muscles, glands, and pain-signal transduction, respectively [1,2,3,4,5,6]. OnabotA is approved in the US for 12 therapeutic and 3 aesthetic indications [5,7]. Per the US Food and Drug Administration (FDA) label, it is approved in adults for the therapeutic treatment of blepharospasm, cervical dystonia (CD), chronic migraine (CM) prophylaxis, upper and lower limb spasticity, neurogenic detrusor overactivity (NDO), overactive bladder (OAB), strabismus, and primary axillary hyperhidrosis. Pediatric indications include spasticity and NDO. OnabotA is also approved for aesthetic applications, including glabellar lines, forehead lines, and lateral canthal lines [7].

The efficacy and safety profile of onabotA for individual FDA-approved therapeutic indications is well established across multiple controlled clinical trials and real-world observational studies [8,9,10,11,12,13,14,15,16]. In general, it is well tolerated over repeated treatments, and adverse events are typically mild, transient, and local in nature [7]. Patients may have comorbid conditions in which onabotA injection is used, leading to multi-indication use. The prescribing information recommends a maximum onabotA cumulative dose of 400 units (U) within a 3-month period when treating adult patients for one or more indications [7]. However, guidance on dose and treatment intervals for multi-indication use are not provided.

Since there are limited data on multi-indication use, providers may have concerns around safety, especially when using higher doses, dosing intervals between treatments of different indications, and the risk of immunogenicity [7,17]. Due to these constraints, treatment with onabotA for concomitant indications may be postponed. This delay can adversely affect patient care and quality of life. Additionally, barriers to access, including a payer’s denial of same-day treatment for multiple indications, mandates on the time between treatments, and treatments exceeding the recommended dose, could result in significant costs and logistical burdens for the patient and provider [18,19]. Patients that are required by payers to receive treatments for multiple indications on separate days may have to make multiple office visits, even when multiple indications are treated by the same provider. This can be particularly challenging for patients who travel from long distances and/or with limited mobility who require a caregiver.

SYNCHRONIZE was a multicenter, retrospective, chart-review study conducted in the US that aims to evaluate the real-word safety profile of onabotA use for adult patients who received treatment for ≥2 therapeutic indications within a 3-month interval. The study assessed treatment-emergent adverse events (TEAEs) following onabotA treatment for multiple indications, a lack of effects as determined via a clinical objective measurement, and utilization in the overall study population and across eleven different treatment-combination groups (TCGs), further stratified by cumulative 3-month dosing and treatment intervals between multiple indications. Safety was evaluated over each 3-month interval with combination treatments for up to 24 months; this analysis focuses on the primary objective of the study, which is the assessment of TEAEs within approximately 6 months after the first 3-month treatment period. These findings will contribute to the understanding of the real-world safety and utilization of onabotA in patients requiring treatment for concomitant therapeutic indications.

## 2. Results

### 2.1. Distribution of Patients across Treatment-Combination Groups

**Figure 1:**
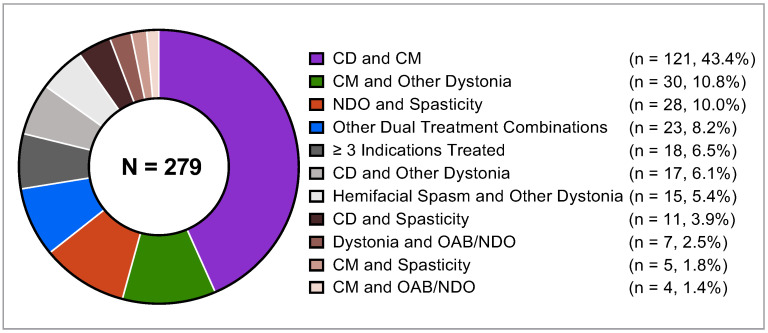
Pie chart depicting the number and proportion of patients within each treatment indication group in the overall patient population (N = 279) analyzed. Refer to Table S1 for a detailed list of the individual therapeutic indications included in each TCG. Abbreviations: CD, cervical dystonia; CM, chronic migraine; n, number of patients in each TCG; N, number of total patients included in the analyses; NDO, neurogenic detrusor overactivity; OAB, overactive bladder; TCG, treatment-combination group.

Among the 279 patients who met study inclusion criteria, there were 44 different TCGs that were consolidated into 11 TCGs for the purposes of these analyses (Figure 1). Detailed descriptions of the original TCGs that make up the consolidated 11 TCGs included in the analyses are available in Table S1. Most patients were treated with onabotA for two different therapeutic indications (261/279; 93.5%) and the remaining 18 patients were treated for ≥3 different therapeutic indications (6.5%). The most common TCG in the analysis population was the CD and CM combination (121/279; 43.4%). The most common TCG with ≥3 indications was CD, CM, and oromandibular dystonia (5/18; 27.8%) (Table S1). Other TCGs included in these analyses were CM and other types of dystonia (30/279; 10.8%) and NDO and spasticity (28/279; 10.0%) (see all consolidated TCGs in Figure 1). Two patients, one in the CD and other dystonia group and one in the hemifacial spasm and other dystonia group, also received concomitant onabotA for aesthetic purposes.

### 2.2. Patient Demographic and Clinical Characteristics

**Figure 2:**
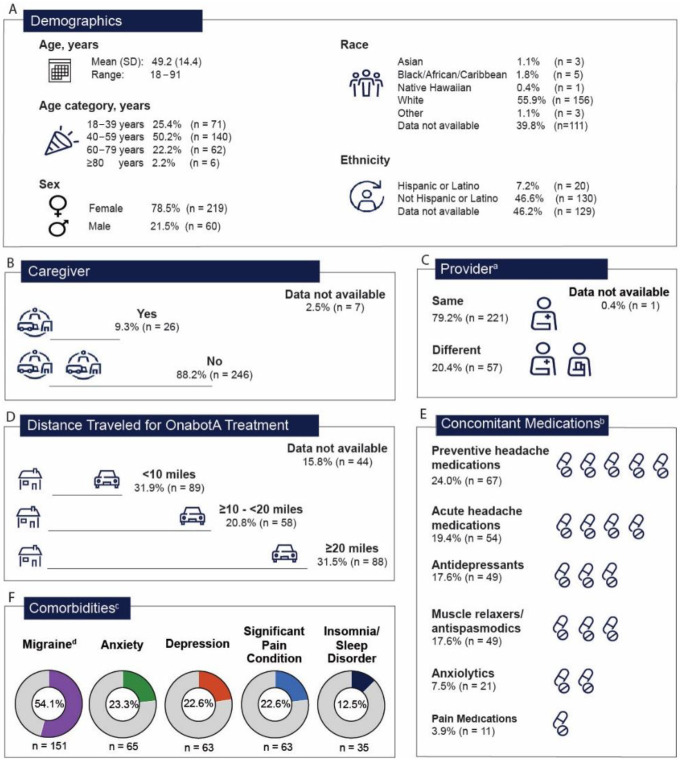
Demographics and patient characteristics within the overall patient population (N = 279) analyzed. (**A**) Demographics; (**B**) caregiver information; (**C**) provider information; (**D**) distance traveled to receive onabotA treatment; (**E**) concomitant medications; (**F**) comorbidities. ^a^ The provider for the second onabotA treatment was the same or different from the first onabotA treatment. ^b^ Baseline concomitant medications and comorbidities were counted at the patient level. ^c^ Baseline comorbidities were defined as present before the index date. ^d^ All patients in this analysis were treated for chronic migraine. Abbreviations: OnabotA, onabotulinumtoxinA; SD, standard deviation.

Baseline characteristics, overall (Figure 2A) and across the eleven consolidated TCGs (Table S2), generally reflected the respective known individual therapeutic indication treated. The mean age in the overall-analysis patient population was 49.2 years (standard deviation [SD]; 14.4), with half of all patients aged between 40 and 59 years (140/279; 50.2%). The majority were female (219/279; 78.5%), and slightly more than half were White (156/279; 55.9%); data were not available on ethnicity for 46.2% of the patients (129/279). Similar demographic patterns were observed across all TCGs at the baseline (Table S2).

A total of 26 patients reported having a caregiver (9.3%) (Figure 2B). Of note, in the following TCGs that included spasticity as an indication, a large proportion of patients reported having a caregiver: NDO and spasticity (11/28; 39.3%), CD and spasticity (3/11; 27.2%), and CM and spasticity (2/5; 40.0%) (Table S2). The majority (221/279; 79.2%) of the overall-analysis population were treated by the same provider for concomitant treatments for multiple indications with onabotA (Figure 2C), which was consistent across all TCGs, except those combination groups that had NDO and OAB as part of their TCGs (Table S2). All patients in the NDO and spasticity TCG (28/28; 100%), as well as dystonia and OAB/NDO TCG (7/7; 100%), and most in the CM and OAB/NDO (3/4; 75%) TCG had a different provider for their subsequent onabotA treatment for a different indication within a 3-month interval (Table S2). Many patients in the overall patient population traveled a long distance for treatment (≥10 to <20 miles [58/279; 20.8%] and ≥20 miles [88/279; 31.5%]), highlighting the potential burden of treating multiple indications on separate days (Figure 2D).

Overall, common comorbidities at the baseline included migraine (151/279; 54.1%), anxiety (65/279; 23.3%), depression (63/279; 22.6%), and a significant pain condition (63/279; 22.6%) (Figure 2E). This trend was broadly consistent across TCGs, except that multiple sclerosis was also common in groups that included NDO as an indication (i.e., NDO and spasticity: 8/28; 28.6%) (Table S3). The most common concomitant medications in the overall-analysis population and across TCGs are highlighted in Figure 2E and Table S3, respectively. In the overall population, common concomitant medications (≥10%) included preventive headache medications (67/279; 24.0%), acute headache medications (54/279; 19.4%), antidepressants (49/279; 17.6%) and muscle relaxers/antispasmodics (49/279; 17.6%) (Figure 2F). This trend was consistent across the TCGs, except that oral medications for urinary incontinence were also common in TCGs that included OAB/NDO as an indication (i.e., NDO and spasticity group: 5/28; 17.9%) (Table S3).

### 2.3. Real-World Treatment Utilization, Cumulative Dose, and Treatment Interval

**Figure 3:**
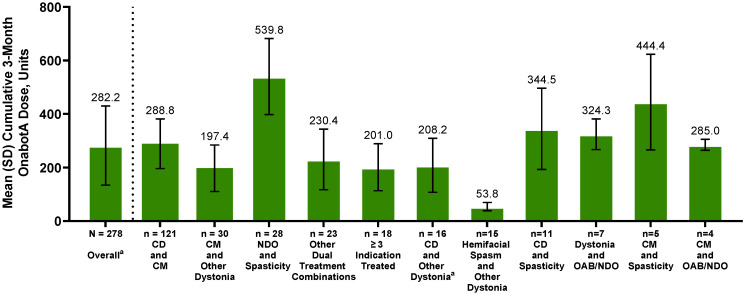
Total cumulative 3-month onabotA dosage overall and across consolidated treatment-combination groups. Refer to Table S1 for a detailed list of the individual therapeutic indications included in each treatment indication combination group. ^a^ One patient in the CD and other dystonia group was missing onabotA dose data and was not included in the analyses for dosing. Abbreviations: CD, cervical dystonia; CM, chronic migraine; NDO, neurogenic detrusor overactivity; OAB, overactive bladder; onabotA, onabotulinumtoxinA; SD, standard deviation.

The overall total 3-month cumulative mean (SD) dose of onabotA administered to treat multiple therapeutic indications was 282.2 (147.8) U (Figure 3). The TCG with the lowest mean (SD) dose was hemifacial spasm and other dystonia at 53.8 (15.4) U, and highest mean dose was in the NDO and spasticity group, 539.8 (142.1) U. The range of total 3-month cumulative dose data presented in Figure 3 depicts the variation in onabotA dosing across different TCGs.

When analyzed across specific dosage range categories in the overall patient population (Table 1), the most frequent 3-month cumulative onabotA dosage administered was ≥200 to <400 U in half of patients (140/278; 50.4%), followed by <200 U (86/278; 30.9%) and ≥400 to <600 units (34/278; 12.2%). A small proportion of patients (18/278; 6.5%) had a cumulative dose that was ≥600 U. Notable differences in the 3-month cumulative dosage categories administered were reported across TCGs. Most patients in the NDO and spasticity group had a cumulative 3-month onabotA dosage that was ≥400 U (24/28; 85.7%) and all patients in the hemifacial spasm and other dystonia group (15/15; 100.0%) had a cumulative 3-month dosage that was <200 U. Only 3 patients had a cumulative 3-month dosage that was ≥800 U, and all were in the NDO and spasticity group (Table 1).

When analyzed across treatment intervals for multiple indications (defined as the difference from the date of the first onabotA treatment and the date at which the patient received the last treatment with onabotA for a different indication) within a 3-month period, most patients in the overall patient population received their cumulative dose of onabotA for multiple different indications within an interval that was ≤24 h (174/279; 62.4%) (Table 2). A total of 27 (9.7%) and 78 (28.0%) patients received a cumulative dose of onabotA within >24 h to 14 days and ≥14 days, respectively. The treatment interval for multiple indications varied across TCGs according to the individual indication treated (Table 2). The cumulative mean (SD) dose administered ≤24 h (on the same day or on consecutive days) for multiple indications was 218.4 (106.7) U; the mean (SD) dose for patients who received their cumulative dose between 2 and 13 days apart and across 14 or more days apart was 418.1 (155.1) U and 376.7 (142.8) U, respectively.

### 2.4. Safety Overview

Safety data are presented in Table 3 as TEAEs assessed within approximately 6 months post-index date, which is the date of the last onabotA treatment for a different indication within the 3-month treatment period analyzed. Across all TCGs, 28.7% (80/279) of patients had ≥1 TEAE. The proportion of patients with ≥1 TEAE varied across TCGs and ranged from 17.4% (4/23) in the other dual treatment combinations group to 60.7% (17/28) in the NDO and spasticity group; in this group, the TEAE incidence was mostly driven by urinary tract infections (UTIs, 11/28; 39.3%). Among the most prevalent TCG, the CD and CM group, 18.2% (22/121) of patients had ≥1 TEAE reported (see a detailed list of TEAE incidence across TCGs in Table 3).

Across all patients included in this analysis, the most common (≥ 2%) TEAEs reported were UTI (16/279; 5.7%), neck pain (14/279; 5.0%), headache (12/279; 4.3%), migraine (10/279; 3.6%), and muscular weakness (6/279; 2.2%) (a list of TEAEs reported in >1% of patients is detailed in Table 3). Most instances of UTIs occurred following treatment for urological conditions. Most of the TEAEs related to headache and migraine occurred after treatment of CM and/or CD, and most TEAEs related to muscular weakness followed treatment for CD. Dysphagia, a common adverse event associated with the treatment of CD [7,15], was reported in four patients (1.4%), including three patients with CD and one patient in the NDO and spasticity group. In the NDO and spasticity patient, dysphagia occurred more than 14 days after onabotA administration and was deemed by the investigator to be unrelated to onabotA treatment. This patient continued treatment with onabotA (Table 3). There were no reported TEAEs considered to be consistent with a possible distant spread of the toxin. 

**Figure 4:**
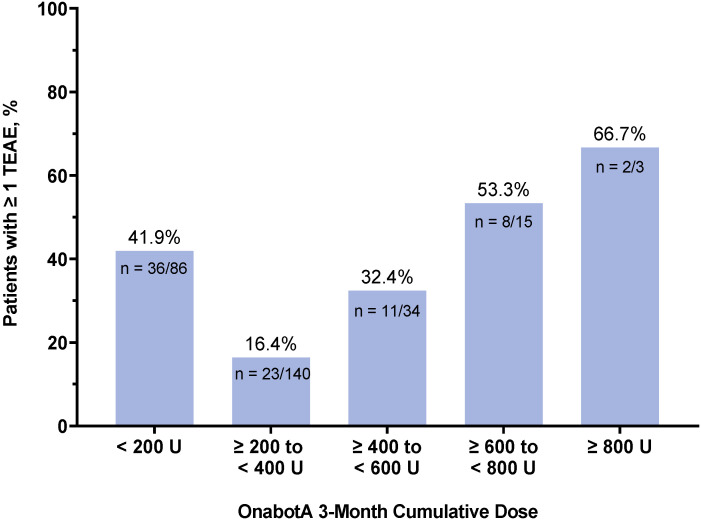
The overall incidence of TEAEs across cumulative 3-month onabotA dose categories. The 3-month interval for calculating cumulative dosage is defined as the first onabotA treatment date plus 3 months. One patient in the CD and other dystonia group was missing treatment dose data and was not included in these analyses. Abbreviations: n, number of patients with ≥1 TEAE over the total number of patients in each dose category; onabotA, onabotulinumtoxinA; TEAE, treatment-emergent adverse event; U, units.

The percentage of patients with ≥1 TEAE across 3-month cumulative dose categories is displayed for the overall patient population in Figure 4 and by TCG in Table S4. No apparent relationship was observed between the TEAE incidence and the onabotA treatment dose categories. The percentage of patients with ≥1 TEAE was slightly higher in the dose categories of ≥600 to <800 U (8/15; 53.3%) and ≥800 U (2/3; 66.7%) (Figure 4); this may be because of smaller sample sizes in the higher dose categories and the recorded TEAE of UTIs associated with the treatment of NDO/OAB, which were more commonly treated in higher dose categories (Table 1). The smallest proportion of patients reporting ≥1 TEAEs (23/140; 16.4%) was in the dose category with the highest proportion of patients (≥200 to <400 units: 140/278; 50.4%) (Table 1 and Figure 4). The most common TEAEs among these patients were headache (6/140; 4.3%) and neck pain (4/140; 2.9%) (Table S5), mostly associated with the treatment of CM and/or CD, since most patients with these indications were treated for those conditions. Overall, TEAEs reported in these dose categories were related to the site of injection. Other common TEAEs reported in ≥1% of patients across dose categories are presented in Table S5.

**Figure 5:**
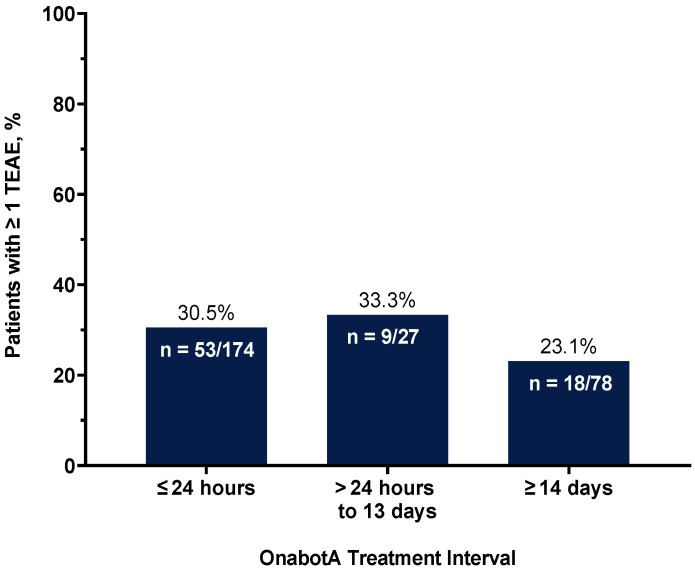
Overall incidence of TEAEs across onabotA treatment intervals between multiple indications within a 3-month treatment period for N = 279 patients. The treatment interval was calculated as the difference between the date of the first qualifying onabotA treatment and the date of the last qualifying onabotA treatment during the primary period; ≤24 h = multiple indications treated on the same day or on two consecutive days; >24 h to 13 days = multiple indications treated between 2 and 13 days apart; ≥14 days = multiple indications treated 14 or more days apart. Abbreviations: n, number of patients with ≥1 TEAE over total number of patients in each treatment interval; onabotA, onabotulinumtoxinA; TEAE, treatment-emergent adverse event.

The percentage of patients with ≥1 TEAE across treatment intervals between multiple indications in the overall patient population is displayed in Figure 5 and by TCG in Table S6. Among the patients who had the most common treatment interval of ≤24 h, 30.5% (53/174) of patients had ≥1 TEAE. The incidence of TEAEs varied across treatment intervals: 33.3% in the >24 h to 13 days and 23.1% in the ≥14 days intervals. There was no evident association between TEAE incidence and treatment intervals (Figure 5). A detailed list of the proportion of patients with ≥1 TEAE reported across each treatment interval stratified by TCG is depicted in Table S6. The most common TEAEs in the ≤24 h interval were neck pain (14/174; 8.1%), followed by headache (11/174; 6.3%), migraine (8/174; 4.6%), and muscular weakness (6/174; 3.5%) (Table S7). These were mostly associated with the treatment of CD and CM since most patients were treated within 24 h for these conditions. Neck pain accounted for 40.7% (11/27) of TEAEs reported in the >24 h to 13 days interval, mostly associated with the treatment of CM, and UTI accounted for 12.8% (10/78) of TEAEs reported in the ≥14 days interval, mostly associated with the treatment of urological conditions. A detailed list of other specific TEAEs reported in ≥1% of patients across treatment intervals is depicted in Table S7.

There were 2 patients among the 279 in the analysis population who also received aesthetic onabotA concurrent with therapeutic indications. Among these patients, there was one reported TEAE within 6 months post-index date. Eyelid ptosis was reported for one patient in the hemifacial spasm and other dystonia group.

### 2.5. Assessment of Lack of Effect and Treatment Discontinuation

There were no patients with a lack of effect documented in the medical record as determined by clinical objective measurement (i.e., brow, frontalis, or extensor digitorum brevis test) in the overall-analysis population. Serum samples were not collected to specifically assess antibody formation.

The documented discontinuation rate for multi-indication treatment with onabotA over the study period was low (5/279; 1.8%); one patient in the NDO and spasticity group discontinued treatment due to adverse events. Another patient in the NDO and spasticity group discontinued treatment for NDO due to augmentation cystoplasty (a typical surgery in the treatment of NDO) but continued to receive onabotA treatment for spasticity. One patient in the CD and CM group rejected future treatments, one patient in the CM and other dystonia group discontinued treatment due to pregnancy, and for one patient in the CM and spasticity group, the physician determined that the treatment was no longer necessary. Across all combination groups, no patient experienced a documented discontinuation of treatment due to insurance restrictions or a lack of effect.

## 3. Discussion

There are limited data available on the safety and utilization of onabotulinumtoxinA (onabotA) when treating patients for multiple indications, which can often lead to delayed or reduced treatment and potentially affect optimal patient care. The purpose of SYNCHRONYZE, a multicenter, retrospective, chart-review study, was to examine the real-world utilization and safety profile of onabotA following treatment in adults for at least two different therapeutic indications within a 3-month interval. To our knowledge, SYNCHRONIZE was the largest observational study to date to investigate the safety of onabotA across multiple therapeutic-indication combinations by reporting the incidence and type of treatment-emergent adverse events (TEAEs), further stratified by indication groups, 3-month cumulative dosing categories, and treatment intervals for multiple indications. The results of this study demonstrated no new safety signals with multi-indication use of onabotA, with TEAEs generally consistent with the known safety profile of individual indications treated and mostly related to the site of injection. The dosing and treatment interval data from this study and the safety profile associated with these results may inform the administration of onabotA in clinical practice (see summary in Audiovisual S1).

In SYNCHRONIZE, the most common treatment-combination group (TCG) was made up of patients (43.4%) who were treated for the dual therapeutic indication of cervical dystonia (CD) and chronic migraine (CM). The high prevalence of this combination reported is consistent with the increasing recognition of the association of CD with CM [20,21,22,23,24]. Clinicians should be aware of coexisting conditions and a cumulative onabotA dose, especially when conditions have overlapping muscles that are dosed for different indications. As the spasticity and neurogenic detrusor overactivity (NDO) combination group was the third most common TCG in this study, urologists and clinicians who treat spasticity and movement disorders should also consider coordinating treatment in medical practice. This study may improve the knowledge of healthcare providers about the diverse and concomitant uses of onabotA outside of their specific areas of expertise and highlight the importance of inquiring about the concomitant use of botulinum toxins with patients. This study emphasizes the significance of effective communication and collaboration among clinicians, as most patients were dosed for different indications within 14 days of each other. Coordination between specialties, such as urologists and physiatrists, is important to manage the timing and dosing of onabotA injections appropriately. By doing so, practitioners can track cumulative dosing, monitor adverse events, and enhance overall treatment outcomes.

This study showed that many patients (31.5%) traveled long distances (≥20 miles) to see a clinician for their treatment, underscoring the burden patients experience when receiving treatment for multiple indications and if they are mandated by their payer to receive treatments on different days. Most patients (62.4%) in this study received their cumulative dosages for multiple indications within 24 h of each other, particularly patients (86.7%) treated concomitantly for CD and CM. Constraints related to insurance coverage may prevent same-day scheduling for multiple indications, which may prevent a clinician from administering the appropriate treatment at the convenience of both the office and the patient. According to some of the physicians’ experiences, when patients travel long distances and are mandated by insurance providers to have treatments on different days, patients routinely stay in nearby hotels and are scheduled for dual treatments within 24 h of each other (i.e., treatment on a Monday afternoon and subsequent treatment on Tuesday morning); however, this subgroup was not further analyzed. The incidence of TEAEs was low across treatment intervals (range: 23.1% to 33.3%), with no apparent relationship observed between the treatment interval for multiple indications and the incidence of TEAEs.

In this study, the total mean 3-month cumulative dose of onabotA administered for multiple indications was 282.2 U. Patients with NDO and spasticity had the highest onabotA cumulative 3-month dosage (539.8 U), and those with hemifacial spasm and other dystonia had the lowest (53.8 U), which is reflective of real-world clinical practice for the individual indication treated. For the majority of patients, the dose administered for multiple indications was within the maximum cumulative dose (400 U) specified in the onabotA US product label [7] (Table 1). Depending on the indication treated, some clinicians in the real-world setting may use higher doses of onabotA than recommended by the US product label to effectively treat patients for their respective indications [25,26,27,28,29,30]. In the current analysis, a proportion of patients (18.7%) received onabotA cumulative doses ≥ 400 U for multiple indications; this was mostly among patients with indications requiring higher doses, such as spasticity. According to a recent safety and dosing report from the Adult Spasticity International Registry (ASPIRE) study on the use of onabotA for adult spasticity [31], most patients received treatment consistent with the maximum cumulative dose (400 U) specified in the onabotA US product label. However, some patients, primarily those with both upper and lower limb spasticity, also received > 400 U onabotA without experiencing any additional safety concerns. This suggests that it is not unreasonable for spasticity patients with concomitant onabotA use for a different indication to exceed the maximum recommended cumulative dose, as the treatment for spasticity alone can reach that limit. The safety results of higher-than-recommended doses in SYNCHRONIZE are consistent with safety data captured in previous studies in which onabotA was used at higher-than-recommended doses [30,31].

Previous studies have shown that repeated onabotA administration to patients over time may lead to the development of neutralizing antibodies [17,32,33,34,35]. Studies have suggested that the development of these antibodies may be linked to shorter intervals between doses (booster injections) and higher doses administered per treatment period [32]. Other reports have linked the occurrence of antibody formation to patient predisposition [17]. While it was not possible to directly evaluate the risk of immunogenicity via antibody assays due to the retrospective nature of this study, there were no indications suggesting immune resistance; no patients had a lack of effect in the medical records as determined by clinical objective measurement documentation, such as a negative frontalis, brow, or extensor digitorum brevis test, nor did any patients have a lack of effect documented as a reason for discontinuation. This is consistent with the low frequency of neutralizing antibody formation following onabotA treatments across multiple indications [7,17].

There was a low proportion of patients (27.8%) who reported ≥1 TEAE within the 6-month post-treatment period for multiple indications in this study. The most reported TEAEs were urinary tract infections (UTIs) in 5.7% of patients, with the highest proportion of patients having overactive bladder (OAB)/NDO as part of their TCG, followed by neck pain (5.0%), with the highest proportion reported for patients treated with onabotA for CD and CM. Headache (4.3%) and migraine (3.6%) were reported, in the majority of cases, by patients whose TCG included CM. These most frequently documented TEAEs were broadly consistent with the onabotA safety profile described in previously published studies for the individual indication treated and that are outlined in the product label [7,31,36,37]. In PREEMPT, the double-blind, randomized controlled studies for CM, 6.7% and 5.5% of patients treated with onabotA for CM experienced neck pain and muscular weakness, respectively [38], while in the current study, in the most commonly reported dual-indication group, CD and CM, neck pain was reported by 8.3% of patients and muscular weakness by 1.7%. UTIs were experienced by 39.3% of patients in the NDO and spasticity group, similar to what was reported in a pooled analysis of two NDO placebo-controlled, phase 3 trials in which UTIs were the most frequently reported TEAE (25%) among subjects treated with onabotA for NDO [11,36,39,40]. In the CD-PROBE clinical trial, dysphagia was reported in 6.4% of CD patients [15], while in the current study, dysphagia was reported in 1.8% of CD patients (3/171).

When providers treat patients for multiple indications that can be treated with onabotA, it is reasonable to utilize a locally acting therapy that can treat both indications to reduce polypharmacy and systemic effects.

### Limitations

Although this study gathered important safety information across a variety of therapeutic indications’ combination groups in real-world clinical practice, several limitations should be considered. Chart reviews rely on information that is already present in medical records; therefore, if certain details, such as TEAEs reported, are not documented in patient charts, it could result in missing or incomplete data. Additionally, not all sites routinely performed a clinical objective test to determine a lack of effect. Treatments administered outside of the electronic medical record (EMR) system may not be reflected in this data set. Site selection may have influenced the reported indications treated, which may limit the generalization of treatment-combination patterns. For example, there was a higher proportion of sites enrolled that treated headache compared to urological conditions.

It is also essential to note that this was a descriptive study intended to provide information on TEAEs reported after onabotA treatment for various therapeutic-indication combinations. No analyses were conducted to determine causality; hence, while the reported TEAEs are associated with onabotA treatment, they may not necessarily have been caused by it. Additionally, the indications treated were heterogeneous, leading to small sample sizes for some therapeutic combination groups, as well as small sample sizes in higher dose categories, which may limit the interpretation of the data. Small sample sizes for patients treated >14 days between indications may also limit the interpretability of safety for extended treatment intervals. It is also important to interpret with caution the safety of using higher-than-recommended doses of onabotA to treat multiple indications concomitantly.

## 4. Conclusions

SYNCHRONIZE described real-world safety in clinical practice when using onabotA for multiple therapeutic indications within a 3-month period. No new safety signals were recorded. Adverse events with multi-indication use were generally consistent with those previously reported for the individual indications and were mostly related to the site of injection; there was no documentation of a lack of effect. There was no apparent relationship between reported TEAEs and treatment interval or 3-month cumulative dose. The findings of this study also underscored patient burden when traveling to see clinicians for multi-indication use. When administering onabotA, providers should consider whether a patient is being treated for multiple indications, coordinate dosing schedules if possible, and alleviate patient burden.

## 5. Materials and Methods

### 5.1. Study Design and Patient Population

**Figure 6:**
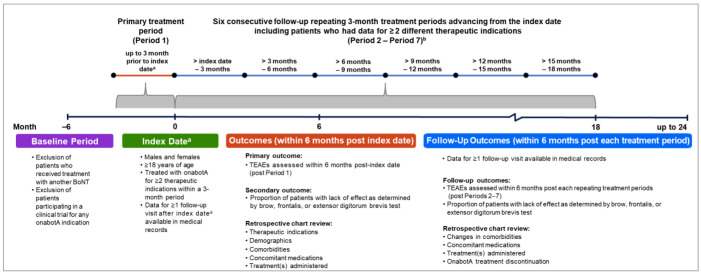
SYNCHRONIZE study design. ^a^ The index date is defined as the date the patient met the study’s inclusion criteria (the date of the last onabotA treatment within the primary period). ^b^ Not all patients had follow-up data to be included in the analyses for all consecutive 7 treatment periods. TEAE assessments were extrapolated from charts approximately 6 months post-each treatment period. Abbreviations: BoNT, botulinum neurotoxin; onabotA, onabotulinumtoxinA; TEAE, treatment-emergent adverse event.

The study design and specific inclusion and exclusion criteria for SYNCHRONIZE are shown in Figure 6. SYNCHRONIZE was a multicenter, retrospective, chart-review study with data collected from January 2010 to January 2022 for patients who were treated with onabotA for ≥2 different therapeutic indications within a 3-month period conducted at 10 clinical sites in the US. All therapeutic indications for onabotA were extracted from charts, including both on-label and off-label indications. The physician (or physicians) was able to treat multiple therapeutic indications at the same visit or at separate visits within a 3-month treatment period. The treatment period included in the analysis corresponds to the date the patient met the study’s inclusion criteria (the date of the last different onabotA indication treated within 3 months of the first onabotA injection for a different indication). Patients were followed up with for additional repeating 3-month treatment periods up to 24 months after the index date if the patients met eligibility criteria at treatment sessions following the primary period (Figure 6).

Institutional review board (IRB) approval was obtained at each participating site before the beginning of data collection. Informed consent was not required, as no identifiable information was provided, and patients were assigned a unique code. No personal identifiers were shared with the sponsor. All data collection was performed in compliance with local ethical and privacy regulations.

The de-identified medical records of patients who met study eligibility criteria with baseline and follow-up data (i.e., follow-up visit, prescription, and scheduled appointment) were recorded. The index date was defined as when the patient met the study’s inclusion criteria (e.g., the date the patient received the second or last onabotA treatment for a different indication in the primary period). Patients treated with onabotA for ≥2 therapeutic indications within a 3-month timeframe of one another during routine clinical practice were included in the analysis. Patients receiving BoNTs other than onabotA for approximately 6 months prior to the index date or at any point during data collection period and patients participating in a clinical trial for any onabotA indication were excluded from the analysis (Figure 6). Patient demographic and clinical characteristics including comorbidities, concomitant medications, and healthcare resource utilizations were recorded during the baseline period (approximately up to 6 months prior to the index date).

TCGs were defined based on the recorded indications for onabotA treatment. The TCGs for a given treatment period were defined as an indication for which onabotA was used as a treatment from the qualifying date of that observation period and from preceding onabotA treatment dates within 3 months (90 days) prior to that date for a different indication. The original 44 TCGs were further consolidated into 11 TCGs for analysis purposes, and they are displayed in Table S1.

### 5.2. Outcome Measurements

OnabotA treatment utilization data and safety were extracted from medical records. Outcome measures were collected during the follow-up period, which was defined from the index date up to approximately 24 months. Outcomes were analyzed for the overall patient population across 11 consolidated TCGs and further stratified into 3 treatment intervals for multiple indications (≤24 h, >24 h to 13 days, and ≥14 days) and 5 dose categories based on cumulative onabotA dose per treatment period (<200 U, ≥200 to <400 U, ≥400 to <600 U, ≥600 to <800 U, and ≥800 U).

The primary objective of SYNCHRONIZE was to assess TEAEs approximately 6 months post-index date for the primary treatment period. TEAEs were collected through chart review and were summarized and reported in all analyses using the *Medical Dictionary for Regulatory Activities* (MedDRA), version 25.1, by preferred terms and system organ class. TEAEs recorded were reported by the patient during post-treatments at a follow-up visit (or visits).

The number and percentage of patients with documented TEAEs within 6 months post-index date for the primary period were reported. The incidence of TEAEs for the treatment intervals and 3-month cumulative dose was also calculated. The total 3-month cumulative dose for patients during the primary period of the study was evaluated by combining the total onabotA dose for all indications treated during the entire 3-month treatment period. Patients receiving cosmetic treatment in conjunction with a therapeutic indication (or indications) and TEAEs documented for these patients were also recorded but not included as an indication in the analyses. For the purposes of this study, an adverse event (AE) was considered any untoward medical occurrence in a patient that may or may not have a causal relationship with the onabotA treatment.

The secondary objective of the study was to evaluate the proportion of patients with a lack of effect, as determined using documentation of a negative frontalis, brow, or extensor digitorum brevis test. These tests can be used to assess whether toxin-neutralizing antibodies may be the cause of a lack of response. The secondary outcome was the assessment of the proportion of patients with a documented lack of effect through an objective measurement, and it was measured within 6 months post-index date for the primary period and during the follow-up treatment periods for which the patient qualified.

### 5.3. Statistical Analyses

Results are provided as descriptive statistics for patient demographics and clinical characteristics and reported as the number and percentage of patients; no inferential statistical analyses were conducted. The overall-analysis population included all enrolled patients who received onabotA to treat ≥2 different therapeutic indications within a 3-month timeframe. The statistical measurements of variables using percentage, frequency, mean, standard deviation (SD), minimum, median, and maximum were used to describe the data. Continuous and discrete variables were also categorized into ranges and described using frequency and percentage distributions. No statistical power calculation was conducted prior to the study; the sample size was based on the available extracted data from the charts.

## Figures and Tables

**Table 1:** Distribution of patients across onabotA cumulative 3-month dose categories stratified by consolidated treatment-combination groups.

		3-Month Cumulative Dose ^a^, *n*/N (%)				
Patient Groups	N	<200 U	≥200–<400 U	≥400–<600 U	≥600–<800 U	≥800 U
Overall ^b^	278	86 (30.9)	140 (50.4)	34 (12.2)	15 (5.4)	3 (1.1)
Treatment-Combination Group						
Cervical Dystonia and Chronic Migraine	121	20 (16.5)	87 (71.9)	13 (10.7)	1 (0.8)	0
Chronic Migraine and Other Dystonia	30	21 (70.0)	8 (26.7)	1 (3.3)	0	0
NDO and Spasticity	28	0	4 (14.3)	10 (35.7)	11 (39.3)	3 (10.7)
Other Dual Treatment Combinations	23	11 (47.8)	9 (39.1)	3 (13.0)	0	0
≥3 Indications Treated	18	10 (55.6)	8 (44.4)	0	0	0
Cervical Dystonia and Other Dystonia ^b^	16	7 (43.8)	9 (56.2)	0	0	0
Hemifacial Spasm and Other Dystonia	15	15 (100)	0	0	0	0
Cervical Dystonia and Spasticity	11	1 (9.1)	6 (54.5)	3 (27.3)	1 (9.1)	0
Dystonia and OAB/NDO	7	0	5 (71.4)	2 (28.6)	0	0
Chronic Migraine and Spasticity	5	1 (20.0)	0	2 (40.0)	2 (40.0)	0
Chronic Migraine and OAB/NDO	4	0	4 (100)	0	0	0

^a^ The 3-month interval for calculating the cumulative dosage for multiple indications is defined as the first onabotA treatment date plus 3 months. ^b^ The dosage was missing for one patient in the CD and other dystonia group and was not included in the analyses for dosing. Abbreviations: CD, cervical dystonia; NDO, neurogenic detrusor overactivity; OAB, overactive bladder; onabotA, onabotulinumtoxinA; U, units.

**Table 2:** Distribution of patients across treatment intervals between multiple indications stratified by treatment-combination groups.

		OnabotA Treatment Interval ^a^, n (%)		
Patient Group	N	≤24 h (n = 174)	>24 h–13 Days (n = 27)	≥14 Days (n = 78)
Overall	279	174 (62.4)	27 (9.7)	78 (28.0)
Treatment-Combination Group				
Cervical Dystonia and Chronic Migraine	121	70 (57.9)	11 (9.1)	40 (33.1)
Chronic Migraine and Other Dystonia	30	26 (86.7)	0	4 (13.3)
NDO and Spasticity	28	3 (10.7)	10 (35.7)	15 (53.6)
Other Dual Treatment Combinations	23	13 (56.5)	3 (13.0)	7 (30.4)
≥3 Indications Treated	18	18 (100)	0	0
Cervical Dystonia and Other Dystonia	17	17 (100)	0	0
Hemifacial Spasm and Other Dystonia	15	15 (100)	0	0
Cervical Dystonia and Spasticity	11	10 (90.9)	0	1 (9.1)
Dystonia and OAB/NDO	7	2 (28.6)	1 (14.3)	4 (57.1)
Chronic Migraine and Spasticity	5	0	1 (20.0)	4 (80.0)
Chronic Migraine and OAB/NDO	4	0	1 (25.0)	3 (75.0)

^a^ The treatment interval is calculated as the difference between the date of the first qualifying onabotA treatment and the date of the last qualifying onabotA treatment for a different indication; ≤24 h = multiple indications treated on the same day or on two consecutive days; >24 h to 13 days = multiple indications treated between 2 to 13 days apart; ≥14 days = multiple indications treated 14 or more days apart. Abbreviations: NDO, neurogenic detrusor overactivity; OAB, overactive bladder; onabotA, onabotulinumtoxinA.

**Table 3:** Overview of TEAEs reported by ≥1 of onabotA-treated patients in the overall population and across treatment-combination groups.

	Overall (N = 279)	CD and CM (n = 121)	CM and Other Dystonia (n = 30)	NDO and Spasticity (n = 28)	Other Dual Treatment Combinations (n = 23)	≥3 Indications Treated (n = 18)	CD and Other Dystonia (n = 17)	Hemifacial Spasm and Other Dystonia (n = 15)	CD and Spasticity (n = 11)	Dystonia and OAB/NDO (n = 7)	CM and Spasticity (n = 5)	CM and OAB/NDO (n = 4)
Patients with ≥1 TEAE within 6 mo post-index date ^a^, n (%)	80 (28.7)	22 (18.2)	8 (26.7)	17 (60.7)	4 (17.4)	10 (55.6)	3 (17.6)	6 (40.0)	3 (27.3)	3 (42.9)	3 (60.0)	1 (25.0)
Specific TEAE ^b^ (in ≥1% of patients) within 6 mo post-index date, n (%)												
Urinary tract infection	16 (5.7)	0	0	11 (39.3)	0	0	0	1 (6.7)	0	2 (28.6)	1 (20.0)	1 (25.0)
Neck pain	14 (5.0)	10 (8.3)	2 (6.7)	0	0	0	1 (5.9)	1 (6.7)	0	0	0	0
Headache	12 (4.3)	6 (5.0)	2 (6.7)	0	0	1 (5.6)	1 (5.9)	1 (6.7)	1 (9.1)	0	0	0
Migraine	10 (3.6)	6 (5.0)	2 (6.7)	0	1 (4.4)	1 (5.6)	0	0	0	0	0	0
Muscular weakness	6 (2.2)	2 (1.7)	1 (3.3)	0	0	1 (5.6)	1 (5.9)	0	1 (9.1)	0	0	0
Eyelid ptosis	5 (1.8)	0	1 (3.3)	0	1 (4.4)	0	0	3 (20.0)	0	0	0	0
Pain in extremity	5 (1.8)	1 (0.8)	1 (3.3)	1 (3.6)	0	1 (5.6)	0	0	1 (9.1)	0	0	2 (50.0)
Dysphagia	4 (1.4)	1 (0.8)	0	1 (3.6) ^c^	0	1 (5.6) ^d^	0	0	0	1 (14.3) ^d^	0	0
Injection site pain	4 (1.4)	0	1 (3.3)	0	0	2 (11.1)	0	0	1 (9.1)	0	0	0
Dizziness	3 (1.1)	1 (0.8)	0	0	0	0	1 (5.9)	0	1 (9.1)	0	0	0
Anxiety	3 (1.1)	1 (0.8)	0	0	0	0	0	0	2 (18.2)	0	0	0
Brow ptosis	3 (1.1)	2 (1.7)	1 (3.3)	0	0	0	0	0	0	0	0	2 (50.0)

^a^ The index date was defined as the date the patient met the study inclusion criteria (i.e., the date of the second or last onabotA treatment within the primary treatment period). ^b^ Patients with ≥1% TEAEs were included. One patient may have reported more than one TEAE. ^c^ A dysphagia patient in the NDO and spasticity group experienced symptoms more than 14 days after onabotA administration that were deemed by the investigator to be unrelated to the treatment, and the patient continued treatment with onabotA. ^d^ Dysphagia patients in the group with ≥3 indications treated and the dystonia and OAB/NDO group also had CD. Abbreviations: CD, cervical dystonia; CM, chronic migraine; NDO, neurogenic detrusor overactivity; OAB, overactive bladder; onabotA, onabotulinumtoxinA; TEAE, treatment-emergent adverse event.

## Data Availability

AbbVie is committed to responsible data sharing regarding the clinical trials we sponsor. This includes access to anonymized, individual, and trial-level data (analysis data sets), as well as other information (e.g., protocols, clinical study reports, or analysis plans), as long as the trials are not part of an ongoing or planned regulatory submission. This includes requests for clinical trial data for unlicensed products and indications. These clinical trial data can be requested by any qualified researchers who engage in rigorous, independent, scientific research, and they will be provided following the review and approval of a research proposal, a statistical analysis plan (SAP), and the execution of a data sharing agreement (DSA). Data requests can be submitted at any time after approval in the US and Europe and after the acceptance of this manuscript for publication. The data will be accessible for 12 months, with possible extensions considered. For more information on the process or to submit a request, visit the following link: https://vivli.org/ourmember/abbvie/ (accessed on 27 September 2024). Then, select “Home”.

## Supplementary Materials

The following supporting information can be downloaded at https://www.mdpi.com/article/10.3390/toxins16100420/s1. Audiovisual S1: Audio and visual summary of SYNCHRONIZE: real-world retrospective safety analysis for patients treated with onabotulinumtoxinA for more than one therapeutic indication; Table S1: Distribution of patients within the consolidated treatment-combination groups and the number of patients within each original treatment-combination group; Table S2: Baseline demographics and patient characteristics across eleven consolidated treatment-combination groups; Table S3: Baseline clinical characteristics across eleven consolidated treatment-combination groups; Table S4: TEAE incidence across onabotA 3-month cumulative dose categories stratified by treatment-combination groups; Table S5: Summary of specific TEAEs stratified by onabotA 3-month cumulative dose categories; Table S6: TEAE incidence across treatment intervals between multiple indications stratified by treatment combinations groups; and Table S7: Summary of specific TEAEs stratified by onabotA treatment interval between multiple indications.

## Abbreviations

CD, cervical dystonia; CM, chronic migraine; HCP, health care provider; NDO, neurogenic detrusor overactivity; onabotA, onabotulinumtoxinA; OAB, overactive bladder; TEAE, treatment-emergent adverse events; TCG, treatment-combination group; U, units.
