# Supplementary material for: SYNCHRONIZE: Real-World Retrospective Safety Analysis of Patients Treated with OnabotulinumtoxinA for More than One Therapeutic Indication
Source: Toxins (Basel). 2024 Sep 29;16(10):420. doi: 10.3390/toxins16100420 (PMC11511055; doi:10.3390/toxins16100420)
Supplement: Supplementary file 1 [file toxins-16-00420-s001.zip › toxins-3207828-supplementary.pdf]

Supplementary Materials: SYNCHRONIZE: Real-World Retrospective Safety Analysis of Patients Treated with OnabotulinumtoxinA for More than One Therapeutic Indication

Grace Forde, Benjamin M. Brucker, Kimberly Becker Ifantides, Atul T. Patel, Angeli Mayadev, Theodore Brown, Ziyad Ayyoub, Kenneth Martinez, Ritu Singh, Mariana Nelson, Simona Battucci, Irina Yushmanova, Ahunna Ukah and Christopher Rhyne

Table S1: Distribution of patients within the consolidated treatment combination groups and the number of patients within each original treatment combination group.

**Consolidated TCG[a], n (%)**	**Overall N = 279**
**Cervical Dystonia and Chronic Migraine**	**121 (43.4)**
**Chronic Migraine and Other Dystonia**	**30 (10.8)**
Chronic Migraine and Oromandibular Dystonia (n = 16)	
Blepharospasm and Chronic Migraine (n = 9)	
Chronic Migraine and Focal Dystonia (n = 5)	
**NDO and Spasticity**	**28 (10.0)**
**Other Dual Treatment Combinations**	**23 (8.2)**
Chronic Migraine and Hemifacial Spasm (n = 4)	
Chronic Migraine and Hyperhidrosis (n = 4)	
Cervical Dystonia and Hemifacial Spasm (n = 2)	
Spasticity and Sialorrhea (n = 2)	
Adductor Spasmodic Dysphonia and Cervical Dystonia (n = 1)	
Blepharospasm and Focal Dystonia (n = 1)	
Blepharospasm and Oromandibular Dystonia (n = 1)	
Blepharospasm and Spasticity (n = 1)	
Chronic Migraine and Gastroparesis (n = 1)	
Chronic Migraine and Neck and Jaw Pain (n = 1)	
Chronic Parotitis and Orofacial Dyskinesia (n = 1)	

Hyperhidrosis and Spasticity (n = 1)	
Myofascial Pain Dysfunction Syndrome and Bruxism (n = 1)	
Myositis of Rectum and Pelvic pain (n = 1)	
Overactive Bladder and Pelvic Pain (n = 1)	
**≥ 3 Indications Treated**	**18 (6.5)**
Cervical Dystonia and Chronic Migraine and Oromandibular Dystonia (n = 5)	
Blepharospasm and Oromandibular Dystonia and Cervical Dystonia (n = 2)	
Cervical Dystonia and Blepharospasm and Chronic Migraine (n = 2)	
Chronic Migraine and Spasticity and Cervical Dystonia (n = 2)	
Blepharospasm and Cervical Dystonia and Oromandibular Dystonia and Chronic Migraine (n = 1)	
Blepharospasm and hemifacial spasm and oromandibular dystonia (n = 1)	
Blepharospasm and spasticity and cervical dystonia (n = 1)	
Cervical Dystonia and Blepharospasm and Hemifacial Spasm (n = 1)	
Chronic Migraine and Blepharospasm and Oromandibular Dystonia (n = 1)	
Hyperhidrosis and Oromandibular Dystonia and Chronic Migraine (n = 1)	
Spasticity and Cervical Dystonia and Chronic Migraine and Oromandibular Dystonia (n = 1)	
**Cervical Dystonia and Other Dystonia**	**17 (6.1)**
Cervical Dystonia and Blepharospasm (n = 6)	
Cervical Dystonia and Oromandibular Dystonia (n = 6)	
Cervical Dystonia and focal Dystonia (n = 3)	
Cervical Dystonia and Essential Tremors (n = 1)	
Cervical Dystonia and Tardive Dyskinesia (n = 1)	
**Hemifacial Spasm and Other Dystonia**	**15 (5.4)**
Blepharospasm and Hemifacial Spasm (n = 14)	
Oromandibular Dystonia and Hemifacial Spasm (n = 1)	
**Cervical Dystonia and Spasticity**	**11 (3.9)**
**Dystonia and OAB/NDO**	**7 (2.5)**
Cervical Dystonia and NDO (n = 4)	
OAB and Focal Dystonia (n = 3)	

**Chronic Migraine and Spasticity**	**5 (1.8)**
**Chronic Migraine and OAB/NDO**	**4 (1.4)**
Chronic Migraine and NDO (n = 3)	
Chronic Migraine and OAB (n = 1)	

[a] 44 different original TCGs were consolidated into 11 recoded TCGs for analyses purposes.
Abbreviations: n, number of patients in each TICG; N, number of total patients included in the analysis; NDO, neurogenic detrusor overactivity; OAB, overactive bladder; TCG, treatment combination group.

Table S2: Baseline demographics and patient characteristics across eleven consolidated treatment combination groups.

Parameter at baseline[a]	Overall (N = 279)	CD and CM (n = 121)	CM and Other Dystonia (n = 30)	NDO and Spasticity (n = 28)	Other Dual Treatment Combinations (n = 23)	≥3 Indications Treated (n = 18)	CD and Other Dystonia (n = 17)	Hemifacial Spasm and Other Dystonia (n = 15)	CD and Spasticity (n = 11)	Dystonia and OAB/NDO (n = 7)	CM and Spasticity (n = 5)	CM and OAB/NDO (n = 4)
**Age (years)**												
Mean (SD)	49.2 (14.4)	46.2 (12.5)	47.6 (10.9)	51.1 (15.5)	47.8 (13.0)	48.3 (16.0)	62.2 (14.9)	58.7 (15.2)	44.5 (17.4)	64.9 (16.2)	44.0 (12.9)	53.0 (17.6)
Median (Min-Max)	48 (18-91)	46 (18-79)	45 (27-70)	51 (20-78)	43 (31-79)	47 (20-81)	62 (34-84)	57 (35-91)	40 (25-74)	66 (34-84)	47 (29-60)	56 (30-70)
**Categorical age (years), n (%)**												
18-39	71 (25.4)	34 (28.1)	6 (20.0)	8 (28.6)	6 (26.1)	6 (33.3)	1 (5.9)	1 (6.7)	5 (45.5)	1 (14.3)	2 (40.0)	1 (25.0)
40-59	140 (50.2)	70 (57.9)	19 (63.3)	11 (39.3)	12 (52.2)	7 (38.9)	6 (35.3)	8 (53.3)	3 (27.3)	1 (14.3)	2 (40.0)	1 (25.0)
60-79	62 (22.2)	17 (14.1)	5 (16.7)	9 (32.1)	5 (21.7)	4 (22.2)	8 (47.1)	4 (26.7)	3 (27.3)	4 (57.1)	1 (20.0)	2 (50.0)
≥80	6 (2.2)	0	0	0	0	1 (5.6)	2 (11.8)	2 (13.3)	0	1 (14.3)	0	0
**Sex[b], n (%)**												
Male	60 (21.5)	21 (17.4)	5 (16.7)	10 (35.7)	8 (34.8)	5 (27.8)	3 (17.6)	3 (20.0)	2 (18.2)	2 (28.6)	1 (20.0)	0
Female	219 (78.5)	100 (82.6)	25 (83.3)	18 (64.3)	15 (65.2)	13 (72.2)	14 (82.4)	12 (80.0)	9 (81.8)	5 (71.4)	4 (80.0)	4 (100.0)
**Race, n (%)**												
Asian	3 (1.1)	0	0	1 (3.6)	1 (4.3)	0	0	0	0	1 (14.3)	0	0
Black/African/ Caribbean	5 (1.8)	0	0	2 (7.1)	1 (4.3)	0	0	1 (6.7)	0	1 (14.3)	0	0
Native Hawaiian/Pacific Islander	1 (0.4)	1 (0.8)	0	0	0	0	0	0	0	0	0	0

White	156 (55.9)	73 (60.3)	10 (33.3)	19 (67.9)	17 (73.9)	3 (16.7)	12 (70.6)	0	8 (72.7)	5 (71.4)	5 (100)	4 (100)
Other	3 (1.1)	1 (0.8)	0	2 (7.1)	0	0	0	0	0	0	0	0
Data not available	111 (39.8)	46 (38.0)	20 (66.7)	4 (14.3)	4 (17.4)	15 (83.3)	5 (29.4)	14 (93.3)	3 (27.3)	0	0	0
**Ethnicity, n (%)**												
Hispanic or Latino	20 (7.2)	3 (2.5)	2 (6.7)	9 (32.1)	2 (8.7)	0	0	0	3 (27.3)	0	0	1 (25.0)
Not Hispanic or Latino	130 (46.6)	61 (50.4)	9 (30.0)	18 (64.3)	11 (47.9)	3 (16.7)	8 (47.1)	1 (6.7)	4 (36.4)	7 (100.0)	5 (100.0)	3 (75.0)
Data not available	129 (46.2)	57 (47.1)	19 (63.3)	1 (3.6)	10 (43.5)	15 (83.3)	9 (52.9)	14 (93.3)	4 (36.4)	0	0	0
**Provider[c], n (%)**												
Same	221 (79.2)	114 (94.2)	26 (86.7)	0	19 (82.6)	18 (100)	17 (100)	15 (100)	9 (81.8)	0	3 (60.0)	0
Different	57 (20.4)	7 (5.8)	4 (13.3)	28 (100)	4 (17.4)	0	0	0	2 (18.2)	7 (100)	2 (40.0)	3 (75.0)
Data not available	1 (0.4)	0	0	0	0	0	0	0	0	0	0	1 (25.0)
**Caregiver, n (%)**												
Yes	26 (9.3)	0	1 (3.3)	11 (39.3)	2 (8.7)	3 (16.7)	0	1 (6.7)	3 (27.3)	3 (42.9)	2 (40.0)	0
No	246 (88.2)	120 (99.2)	29 (96.7)	16 (57.1)	21 (91.3)	15 (83.3)	14 (82.4)	14 (93.3)	8 (72.7)	3 (42.9)	2 (40.0)	4 (100)
Data not available	7 (2.5)	1 (0.8)	0	1 (3.6)	0	0	3 (17.7)	0	0	1 (14.3)	1 (20.0)	0
**Miles traveled to treatment, n (%)**												
<10 miles	89 (31.9)	35 (28.9)	16 (53.3)	8 (28.6)	6 (26.1)	7 (38.9)	2 (11.8)	10 (66.7)	3 (27.3)	1 (14.3)	0	1 (25.0)
≥10 to <20 miles	58 (20.8)	23 (19.0)	1 (3.3)	8 (28.6)	5 (21.7)	4 (22.2)	7 (41.2)	1 (6.7)	5 (45.5)	2 (28.6)	1 (20.0)	1 (25.0)
≥20 miles	88 (31.5)	48 (39.7)	6 (20.0)	6 (21.4)	4 (17.4)	7 (38.9)	6 (35.3)	3 (20.0)	3 (27.3)	3 (42.9)	1 (20.0)	1 (25.0)
Data not available	44 (15.8)	15 (12.4)	7 (23.3)	6 (21.4)	8 (34.8)	0	2 (11.8)	1 (6.7)	0	1 (14.3)	3 (60.0)	1 (25.0)

[a] Baseline period is approximately 6 months prior to index date.
[b] Gender was also evaluated from patients' charts and the results were similar to what was reported for sex.
[c] The provider for the second onabotA treatment is the same or different from the first onabotA treatment.
Abbreviations: CD, cervical dystonia; CM, chronic migraine; NDO, neurogenic detrusor overactivity; OAB, overactive bladder; onabotulinumtoxinA, onabotA; SD, standard deviation.

Table S3: Baseline clinical characteristics across eleven consolidated treatment combinations groups.

Parameter and baseline[a]	Overall (N = 279)	CD and CM (n = 121)	CM and Other Dystonia (n = 30)	NDO and Spasticity (n = 28)	Other Dual Treatment Combinations (n = 23)	≥ 3 Indications Treated (n = 18)	CD and Other Dystonia (n = 17)	Hemifacial Spasm and Other Dystonia (n = 15)	CD and Spasticity (n = 11)	Dystonia and OAB/NDO (n = 7)	CM and Spasticity (n = 5)	CM and OAB/NDO (n = 4)
**Concomitant medications (in ≥ 3%), n (%)**												
Preventative headache medications	67 (24.0)	40 (33.1)	12 (40.0)	5 (17.9)	3 (13.0)	1 (5.6)	1 (5.9)	0	0	2 (28.6)	2 (40.0)	1 (25.0)
Acute headache medications	54 (19.4)	38 (31.4)	12 (40.0)	0	2 (8.7)	2 (11.1)	0	0	0	0	0	0
Muscle relaxers/antispasmodics	49 (17.6)	30 (24.8)	4 (13.3)	4 (14.3)	4 (17.4)	2 (11.1)	1 (5.9)	0	2 (18.2)	1 (14.3)	1 (20.0)	0
Antidepressants	49 (17.6)	30 (24.8)	5 (16.7)	4 (14.3)	3 (13.0)	3 (16.7)	1 (5.9)	0	2 (18.2)	1 (14.3)	0	0
Anxiolytics	21 (7.5)	12 (9.9)	1 (3.3)	2 (7.1)	2 (8.7)	0	2 (11.8)	1 (6.7)	0	1 (14.3)	0	0
Pain medications	11 (3.9)	3 (2.5)	1 (3.3)	3 (10.7)	2 (8.7)	1 (5.6)	0	0	0	1 (14.3)	0	0
Oral urinary incontinence medications	10 (3.6)	1 (0.8)	0	5 (17.9)	0	0	1 (5.9)	0	0	1 (14.3)	0	2 (50.0)
**Comorbidities (in ≥ 1%) , n (%)**												
Migraine	151 (54.1)	89 (73.6)	25 (83.3)	0	7 (30.4)	14 (77.8)	2 (11.8)	5 (33.3)	0	2 (28.6)	5 (100)	2 (50.0)
Anxiety	65 (23.3)	29 (24.0)	12 (40.0)	5 (17.9)	5 (21.7)	7 (38.9)	2 (11.8)	1 (6.7)	0	2 (28.6)	2 (40.0)	0
Significant pain condition	63 (22.6)	23 (19.0)	8 (26.7)	3 (10.7)	2 (8.7)	11 (61.1)	4 (23.5)	4 (26.7)	4 (36.4)	3 (42.9)	0	1 (25.0)
Depression	63 (22.6)	28 (23.1)	11 (36.7)	7 (25.0)	5 (21.7)	4 (22.2)	1 (5.9)	2 (13.3)	0	2 (28.6)	2 (40.0)	1 (25.0)

Insomnia/Sleep disorder	35 (12.5)	15 (12.4)	7 (23.3)	2 (7.1)	1 (4.4)	5 (27.8)	0	2 (13.3)	0	1 (14.3)	2 (40.0)	0
Chronic urinary tract infection	16 (5.7)	0	0	12 (42.9)	1 (4.4)	0	0	0	0	2 (28.6)	0	1 (25.0)
Bruxism	13 (4.7)	3 (2.5)	4 (13.3)	0	2 (8.7)	3 (16.7)	0	1 (6.7)	0	0	0	0
Multiple sclerosis	10 (3.6)	0	0	8 (28.6)	0	0	0	0	0	2 (28.6)	0	0
Fibromyalgia	9 (3.2)	5 (4.1)	2 (6.7)	0	0	2 (11.1)	0	0	0	0	0	0
Temporomandibular joint disorder	9 (3.2)	2 (1.7)	3 (10.0)	0	1 (4.4)	1 (5.6)	0	1 (6.7)	0	0	1 (20.0)	0
Parkinson's disease	9 (3.2)	0	1 (3.3)	1 (3.6)	1 (4.4)	0	3 (17.7)	1 (6.7)	0	2 (28.6)	0	0
Traumatic brain injury	8 (2.9)	3 (2.5)	1 (3.3)	1 (3.6)	1 (4.4)	1 (5.6)	0	0	0	0	1 (20.0)	0
Spinal cord injury	4 (1.4)	0	0	4 (14.3)	0	0	0	0	0	0	0	0
Bipolar disorder	4 (1.4)	2 (1.7)	0	1 (3.6)	1 (4.4)	0	1 (5.6)	0	0	0	0	0

[a] Baseline concomitant medications and comorbidities were counted at the patient-level. Baseline comorbidities defined as present before the index date; baseline period is approximately 6 months before the index date.

Abbreviations: CD, cervical dystonia; CM, chronic migraine; NDO, neurogenic detrusor overactivity; OAB, overactive bladder.

Table S4: TEAE incidence across onabotA 3-month cumulative dose categories stratified by treatment combination groups.

Patients With ≥ 1 TEAE Within 6 Months Post Index Date	Overall N	OnabotA 3-Month Cumulative Dose[a], n (%)									
		n	< 200 U	n	≥ 200 to < 400 U	n	≥ 400 to <600 U	n	≥ 600 to < 800 U	n	≥ 800 U
**Overall[b]**	278	**86**	**36 (41.9)**	**140**	**23 (16.4)**	**34**	**11 (32.4)**	**15**	**8 (53.3)**	**3**	**2 (66.7)**
**Treatment Indication Combination group**											
Cervical Dystonia and Chronic Migraine	121	20	11 (55.0)	87	9 (10.3)	13	2 (15.4)	1	0	0	0
Chronic Migraine and Other Dystonia	30	21	7 (33.3)	8	1 (12.5)	1	0	0	0	0	0
NDO and Spasticity	28	0	0	4	2 (50.0)	10	6 (60.0)	11	7 (63.6)	3	2 (66.7)
Other Dual Treatment Combinations	23	11	4 (36.4)	0	0	0	0	0	0	0	0
≥ 3 Indications Treated	18	10	6 (60.0)	8	4 (50.0)	0	0	0	0	0	0
Cervical Dystonia and Other Dystonia[b]	16	7	1 (14.3)	9	2 (22.2)	0	0	0	0	0	0
Hemifacial Spasm and Other Dystonia	15	15	6 (40.0)	0	0	0	0	0	0	0	0
Cervical Dystonia and Spasticity	11	1	1 (100)	6	2 (33.3)	3	0	1	0	0	0
Dystonia and OAB/NDO	7	0	0	5	2 (40.0)	2	1 (50.0)	0	0	0	0
Chronic Migraine and Spasticity	5	1	0	0	0	2	2 (100)	2	1 (50.0)	0	0
Chronic Migraine and OAB/NDO	4	0	0	4	1 (25.0)	0	0	0	0	0	0

[a] The 3-month interval for calculating cumulative dosage is defined as the first onabotA treatment date plus 3 months.

[b] The dose was missing in 1 patient.

Abbreviations: n, number of patients in each dose category; NDO, neurogenic detrusor overactivity; OAB, overactive bladder; onabotA, onabotulinumtoxinA; TEAE, treatment-emergent adverse event; U, units.

Table S5: Summary of specific TEAEs stratified by onabotA 3-month cumulative dose categories.

	OnabotA 3-Month Cumulative Dose[a,b], n (%)				
	**< 200 U (n = 86)**	**≥ 200 to < 400 U (n = 140)**	**≥ 400 to <600 U (n = 34)**	**≥ 600 to < 800 U (n = 15)**	**≥ 800 U (n = 3)**
**Specific TEAEs (in ≥ 1% patients) within 6 months post index date**					
Neck pain	10 (11.6)	4 (2.9)	0	0	0
Headache	6 (7.0)	6 (4.3)	0	0	0
Migraine	6 (7.0)	3 (2.1)	1 (2.9)	0	0
Eyelid ptosis	5 (5.8)	0	0	0	0
Muscular weakness	4 (4.7)	2 (1.4)	0	0	0
Injection site pain	3 (3.5)	1 (0.7)	0	0	0
Pain in extremity	3 (3.5)	1 (0.7)	1 (2.9)	0	0
Asthenia	2 (2.3)	0	0	0	0
Brow ptosis	2 (2.3)	1 (0.7)	0	0	0
Dry eye	2 (2.3)	0	0	0	0
Upper urinary tract infection	2 (2.3)	0	0	0	0
Dysphagia	1 (1.2)	2 (1.4)	0	1 (6.7)	0
Urinary tract infection	1 (1.2)	3 (2.1)	6 (17.7)	4 (26.7)	2 (66.7)
Anxiety	1 (1.2)	2 (1.4)	0	0	0

[a] The 3-month interval for calculating cumulative dosage is defined as the first onabotulinumtoxinA treatment date plus 3 months.

[b] The dose was missing in one patient.

Abbreviations: n, number of patients in each dose category; onabotA, onabotulinumtoxinA; TEAE, treatment-emergent adverse event; U, units.

Table S6: TEAE incidence across treatment interval between multiple indications stratified by treatment combinations groups.

Patients With ≥ 1 TEAE Within 6 Months Post-Index Date	Overall N	OnabotA Treatment Interval[a], n (%)					
		n	≤ 24 hours	n	> 24 Hours to 13 Days	n	≥ 14 Days
**Overall**	279	174	53 (30.5)	27	9 (33.3)	78	18 (23.1)
**Treatment combination group[a]**							
Cervical Dystonia and Chronic Migraine	121	70	19 (27.1)	11	1 (9.1)	40	2 (5.0)
Chronic Migraine and Other Dystonia	30	26	7 (26.9)	1	0	3	1 (33.3)
NDO & Spasticity	28	3	2 (66.7)	10	7 (70.0)	15	8 (53.3)
Other Dual Treatment Combinations	23	13	2 (15.4)	3	0	7	2 (28.6)
≥3 Indications Treated	18	18	10 (100)	0	0	0	0
Cervical Dystonia and Other Dystonia	17	17	3 (17.7)	0	0	0	0
Hemifacial Spasm and Other Dystonia	15	15	6 (40.0)	0	0	0	0
Cervical Dystonia and Spasticity	11	10	3 (30.0)	0	0	1	0
Dystonia and OAB/NDO	7	2	1 (50.0)	1	0	4	2 (50.0)
Chronic Migraine and Spasticity	5	1	1 (100)	0	0	4	2 (50.0)
Chronic Migraine and OAB/NDO	4	1	0	4	1 (25.0)	3	1 (33.3)

[a] Treatment interval is calculated as the difference between the date of the first qualifying onabotulinumtoxinA treatment and the date of the last qualifying onabotulinumtoxinA treatment; ≤ 24 hours = multiple indications treated on the same day or on two consecutive days; > 24 hours to 13 days = multiple indications treated between 2 to 13 days apart; ≥ 14 days = multiple indications treated 14 or more days apart.

Abbreviations: n, number of patients in each treatment interval; NDO, neurogenic detrusor overactivity; OAB, overactive bladder; TEAE, treatment-emergent adverse event; U, units.

Table S7: Summary of specific TEAEs stratified by onabotA treatment interval between multiple indications.

	**OnabotA Treatment Interval[a], n (%)**		
	**≤ 24 hours (n = 174)**	**> 24 Hours to 13 Days (n = 27)**	**≥ 14 Days (n = 78)**
**Specific TEAEs (in ≥ 1% patients) within 6 months post index date**			
Neck pain	14 (8.1)	11 (40.7)	2 (5.0)
Headache	11 (6.3)	1 (3.7)	0
Migraine	8 (4.6)	0	2 (5.0)
Muscular weakness	6 (3.5)	0	0
Eyelid ptosis	5 (2.9)	0	0
Injection site pain	4 (2.3)	0	0
Anxiety	3 (1.7)	0	0
Brow ptosis	3 (1.7)	0	0
Dizziness	3 (1.7)	0	0
Dysphagia	3 (1.7)	1 (3.7)	0
Pain in extremity	3 (1.7)	1 (3.7)	1 (1.3)
Urinary tract infection	3 (1.7)	3 (11.1)	10 (12.8)

[a] Treatment interval is calculated as the difference between the date of the first qualifying onabotA treatment and the date of the second or last qualifying onabotulinumtoxinA treatment; ≤ 24 hours = multiple indications treated on the same day or on two consecutive days; > 24 hours to 13 days = multiple indications treated between 2 to 13 days apart; ≥ 14 days = multiple indications treated 14 or more days apart.
Abbreviations: n, number of patients in each treatment interval; onabotA, onabotulinumtoxinA; TEAE, treatment-emergent adverse event.
